# Urinary Microbiota Composition in Treatment-Naïve Bladder Cancer: A Case–Control Study with Tumor Invasiveness Stratification

**DOI:** 10.3390/medicina61122240

**Published:** 2025-12-18

**Authors:** Ahmet Kayer, Ata Özen, Ener Çağrı Dinleyici

**Affiliations:** 1 Department of Urology, Faculty of Medicine, Eskisehir Osmangazi University, Eskisehir 26040, Türkiye; ahmtkyr@hotmail.com; 2 Department of Pediatrics, Faculty of Medicine, Eskisehir Osmangazi University, Eskisehir 26040, Türkiye; timboothtr@yahoo.com

**Keywords:** bladder cancer, microbiota, microbiome, urinary microbiota

## Abstract

*Background and Objectives*: Emerging evidence suggests that the genitourinary microbiota may influence the development and progression of urological malignancies, including bladder cancer. This study aimed to characterize the urinary microbiota at diagnosis in patients with bladder cancer and compare findings with healthy controls. *Materials and Methods:* Urine samples were collected from 30 patients with treatment-naïve bladder cancer and 20 age- and sex-matched healthy individuals. Microbiota composition was analyzed using 16S rRNA sequencing, and subgroup comparisons were made between muscle-invasive bladder cancer (MIBC) and non-muscle-invasive bladder cancer (NMIBC). Differentially abundant taxa were identified using linear discriminant analysis effect size (LEfSe) with an LDA threshold > 2 and *p* < 0.05. *Results:* No significant differences were observed in alpha or beta diversity between patients and controls or between MIBC and NMIBC. At the phylum level, *Firmicutes* was dominant in both groups but relatively more abundant in bladder cancer cases. *Enterococcus* was the most abundant genus in the cancer group (35.0%) and especially in MIBC (58.0%), while *Lactobacillus* was more prevalent in healthy controls (19.8% vs. 9.5%). At the species level, *Veillonella dispar* was notably enriched in MIBC cases (70.9%) compared to NMIBC (3.9%). LEfSe analysis revealed significant enrichment of *Ralstonia*, *Microbacterium*, and *Facklamia* in patients with bladder cancer, while *Parvimonas*, *Sneathia*, *Gemella*, and *Acinetobacter guillouiae* were more abundant in controls. *Conclusions:* These findings highlight preliminary microbiota differences associated with bladder cancer and tumor invasiveness; however, the results are exploratory and larger studies are required to evaluate their diagnostic or clinical relevance.

## 1. Introduction

Bladder cancer is the most common malignant tumor of the urinary system and is associated with significant morbidity and mortality worldwide [1]. According to the World Health Organization, approximately 430,000 new cases are diagnosed each year, with men affected four times more often than women and incidence increasing with age [1,2]. Major risk factors include tobacco use and exposure to industrial carcinogens [2,3]. Urothelial carcinoma accounts for over 90% of bladder cancer cases, with less common histological subtypes including squamous cell carcinoma, adenocarcinoma, and sarcomas [4]. The hallmark symptom is painless hematuria with clots [2]. Around 25% of newly diagnosed bladder cancers are muscle-invasive (MIBC), while the majority are non-muscle-invasive (NMIBC) [5,6]. NMIBC has a high recurrence rate (60–70%), and 20% may progress to MIBC [6]. Despite being non-invasive initially, about 50% of NMIBC cases may eventually develop nodal or distant metastasis [7]. Diagnosis relies primarily on cystoscopy and histopathological examination following transurethral resection of the bladder tumor, which also provides therapeutic benefit [8].

In recent years, growing interest has emerged around the human microbiota—the complex ecosystem of bacteria, fungi, and viruses residing on and within the body [9]. With the help of high-throughput DNA sequencing technologies, it is now clear that even urine, once considered sterile, hosts its own distinct microbiota [10]. The composition of the urinary microbiome may be influenced by various diseases, such as urinary tract infections, interstitial cystitis, urinary incontinence, and bladder pain syndrome [10,11]. Studies investigating the role of the urinary microbiota in bladder cancer remain limited and inconclusive [11,12,13]. According to a systematic review of urinary microbiome studies in bladder cancer, beta-diversity and microbial composition differed significantly between cancer patients and controls, while alpha-diversity outcomes varied considerably among studies [14]. The urinary microbiota exhibits distinct patterns across healthy individuals, NMIBC, and MIBC, which may relate to biological processes involved in tumor progression. In addition, emerging evidence suggests that the microbiota may independently influence treatment response, although these represent distinct mechanisms [14].

This study aims to compare the urinary microbiota of patients with bladder cancer to that of healthy individuals, and to explore differences in microbiota diversity and composition according to disease stage (MIBC vs. NMIBC).

## 2. Patient and Methods

This is a single-center prospective case–control study evaluating urinary microbiota profiles in patients with treatment-naïve bladder cancer and age- and sex-matched healthy controls between 26 April 2022, and 7 March 2023, at the Urology Clinic of Eskişehir Osmangazi University Faculty of Medicine. All participants provided written informed consent before enrollment.

Inclusion and Exclusion Criteria: Patients with bladder cancer eligible for the study were aged 40–80 years, had not previously been diagnosed or treated for bladder cancer, and were scheduled to undergo transurethral resection of a bladder tumor following cystoscopic identification of a mass. Healthy individuals aged 40–80 years, with no active urinary, systemic, or oncologic disease and no history of recent medication use, were recruited as the control group. Exclusion criteria for both groups included active urinary tract infection, antibiotic use in the past 8 weeks, body mass index (BMI) > 30 kg/m^2^, use of probiotics, immunosuppressive therapy, presence of systemic immune disorders, previous pelvic radiotherapy, history of any malignancy other than bladder cancer, and age < 40 or >80 years. Histopathological parameters were obtained from routine diagnostic pathology evaluations. Pathological staging was defined according to the TNM system: pTa (non-invasive papillary carcinoma), pT1 (tumor invasion into the lamina propria), and pT2 (tumor invasion into the muscularis propria). Tumor grade was defined using the World Health Organization grading system. The presence of concomitant carcinoma in situ and any variant histology patterns was documented as binary variables. Pathological staging was classified according to standard criteria into muscle-invasive (MIBC) and non-muscle-invasive bladder cancer (NMIBC) [5,6,8].

Urine Sample Collection: Prior to transurethral resection of a bladder tumor and under direct medical supervision, 30 mL of midstream urine was collected into sterile 50 mL falcon tubes. To reduce the risk of contamination, the glans penis or external urethral orifice was cleansed with 10% povidone-iodine before collection. We clarified that healthy individuals underwent urinalysis to exclude asymptomatic infection or hematuria. Samples were immediately stored at −80 °C until processing.

DNA Extraction: Frozen urine samples were thawed on ice, and 2 mL aliquots were transferred into microcentrifuge tubes. Samples were centrifuged at 16,000× *g* for 15 min at 4 °C, and the supernatant was discarded. DNA was extracted from the resulting pellet using the DiaRex^®^ Stool Genomic DNA Extraction Kit (Cat No: SD-0323, Ankara, Türkiye) following an adapted protocol. 250 µL Lysis Buffer was added to the pellet. 15 mg of glass beads and 10 zirconium beads were added, and homogenization was performed at 4000 rpm for 2 × 20 s. 25 µL Proteinase K was added, followed by incubation at 56 °C for 60 min. The sample was centrifuged at 5000× *g* for 5 min, and the supernatant was transferred to a new tube. 200 µL Stool Lysis Buffer was added, followed by incubation at 70 °C for 10 min. 250 µL of absolute ethanol was added, and the lysate was transferred to a silica column. The column was washed according to the kit protocol, and DNA was eluted in 100 µL Elution Buffer.

16S Amplicon Sequencing and Bioinformatic Analysis: The 16S rRNA V3-V4 region was amplified and sequenced on the Illumina MiSeq platform. Pooled libraries cleaned up with specific size selection were applied by following the manufacturer’s protocol (AMPure XP, Beckman Coulter, Brea, CA, USA). After library preparation, the NovoSeq 6000 (Illumina, San Diego, CA, USA) instrument was used to run sequencing. Pair-end Illumina reads (2 × 250) was imported to the QIIME2 environment. Quality clipping, chimera detection, and cleaning of reads implemented through the QIIME2 DADA2 pipeline (q2-dada2, DADA2 v1.30.0). Amplicon Sequence Variants (ASVs) generated by DADA2 were mapped to GreenGenes 13_8 database (http://greengenes.lbl.gov). The Phyloseq v1.46.0 object was created from QIIME2 artifact files in the R 4.4.1 environment using the QIIME22R v0.99.12 package. Alpha diversity assessment, including Chao1, Shannon, and Observed OTUs implemented via the vegan v2.6-6 and microbiome v1.24.0 packages. *p* values between groups were calculated with the Kruskal–Wallis test. Beta diversity analysis, used to assess taxonomic differences between individuals, was calculated based on Jaccard, Bray–Curtis, weighted and unweighted UniFrac, using the vegan v.2.6_6, ape v5.8, and picante v1.8.2 packages. Specific differences between groups were determined by differential abundance analysis with the DESeq2 v1.42.0 R package. Linear discriminant analysis Effect Size (LEFSe) analysis was conducted using the microbiomeMarker v1.3.1 with a significance threshold of LDA > 2.0 and *p* < 0.05. R implementation to identify statistically significant taxonomy.

Statistical analysis: Statistical analyses were performed using the JASP software (JASP Team, 2024; Version 0.19.3). Categorical variables were reported as frequencies and percentages, and continuous variables as means ± standard deviations, as appropriate. Comparisons between groups (bladder cancer vs. control) were conducted using chi-square or Fisher’s exact test for categorical variables and t-tests for continuous variables. A two-tailed *p* < 0.05 was considered significant.

## 3. Results

Patient Demographics: 30 newly diagnosed patients with bladder cancer and 20 healthy individuals have been enrolled. The bladder cancer group included 4 women and 26 men, while the control group included 3 women and 17 men. There were no statistically significant differences between the groups in terms of gender, age (69.4 ± 9.8 vs. 67.6 ± 9.3 years), or body mass index (24.9 ± 3.7 vs. 24.9 ± 3.3 kg/m^2^). However, the proportion of smokers was higher in the bladder cancer group (80%) compared to controls (45%) (*p* = 0.013). Among the bladder cancer cases, tumor stage based on primary pathology was classified as follows: Ta in 50% of patients (n = 15), T1 in 30% (n = 9), and T2 in 20% (n = 6). Based on the TNM classification system, 40% of tumors (n = 12) were categorized as low stage, while 60% (n = 18) were considered high stage. Carcinoma in situ (CIS) was present in 40% of cases (n = 12), and variant histological features were observed in 24% of patients. A detailed comparison of demographic and clinical characteristics between patients with bladder cancer and healthy controls is presented in Table 1.

### 3.1. Urinary Microbiota Composition

**Figure 1:**
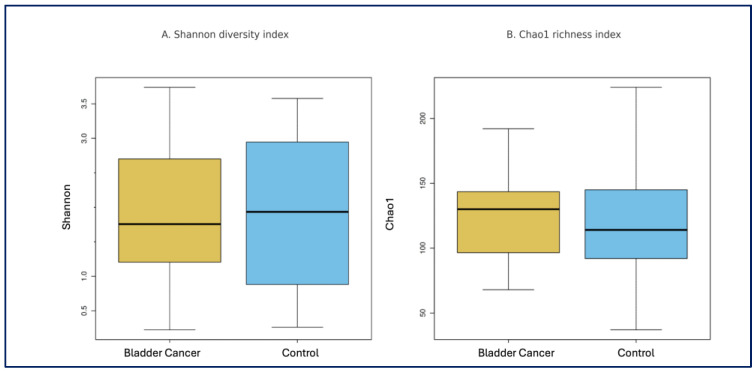
Comparison of alpha-diversity indices between bladder cancer patients and healthy controls. (**A**) Shannon diversity index and (**B**) Chao1 richness index. Boxes represent the interquartile range (IQR), horizontal lines indicate the median, and whiskers extend to 1.5 × IQR.

**Figure 2:**
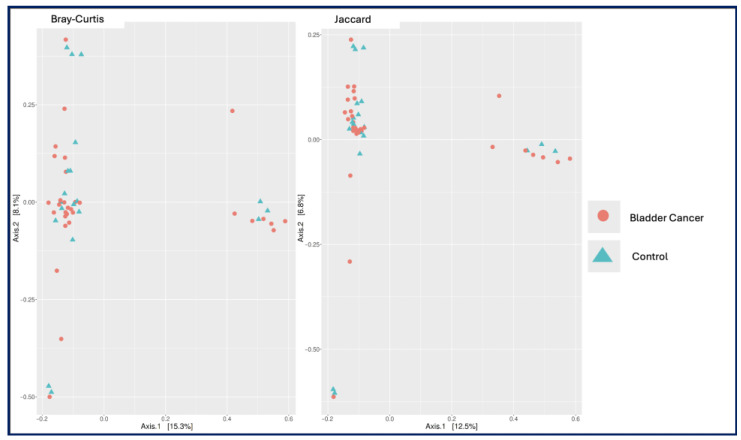
Principal coordinates analysis (PCoA) of urinary microbiota profiles in bladder cancer patients and healthy controls based on Bray–Curtis dissimilarity (**left**) and Jaccard distance (**right**). Each point represents an individual sample, with bladder cancer samples shown in red circles and control samples in blue triangles. Percent variance explained by each axis is indicated in brackets. The plots illustrate overall community structure differences between groups.

Alpha and Beta Diversity: Alpha diversity indices, including Observed OTUs, Chao1, and Shannon index, did not differ significantly between the bladder cancer and control groups (*p* = 0.960, *p* = 0.528, and *p* = 0.880, consecutively) (Figure 1). Similarly, principal coordinate analysis (PCoA) based on Bray–Curtis and Jaccard distances revealed no significant differences in beta diversity between the two groups (*p* = 0.938 and *p* = 0.929; Figure 2).

**Figure 3:**
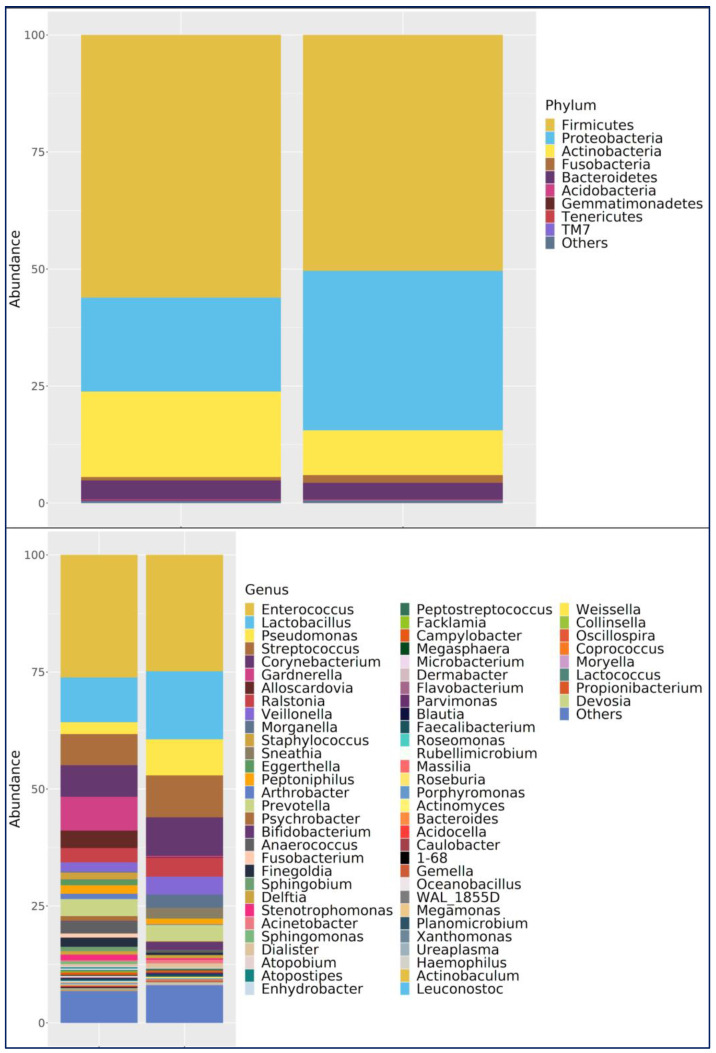
Comparison of urinary microbiota composition between bladder cancer patients (**left**) and healthy controls (**right**) at the phylum level (**top**) and genus level (**bottom**). Stacked bar plots represent relative abundance of major taxa, with low-abundance taxa grouped as “Others.” Differences illustrate the overall community structure between groups.

Phylum Level: In both groups, *Firmicutes* was the dominant phylum. In the bladder cancer group, *Firmicutes* (63.3%), *Actinobacteria* (16.6%), *Proteobacteria* (16.0%), and *Bacteroidetes* (3.4%) were most abundant. In controls, *Firmicutes* (46.3%) and *Proteobacteria* (39.0%) were dominant, followed by *Actinobacteria* (7.6%), *Bacteroidetes* (4.2%), and Fusobacteria (2.3%) (Figure 3).

Genus Level: *Enterococcus* was the most abundant in both the bladder cancer group and the healthy control group. *Enterococcus* was the most abundant genus in the bladder cancer group (35.0%), followed by *Lactobacillus* (9.5%), *Gardnerella* (8.0%), and *Streptococcus* (7.0%). In controls, *Lactobacillus* and *Enterococcus* (both 19.8%) were most abundant, along with *Pseudomonas* (9.9%), *Streptococcus* (7.5%), *Corynebacterium* (5.4%), *Prevotella* (4.6%), and *Ralstonia* (4.0%) (Figure 3).

Species Level: *Lactobacillus iners* was the most abundant species in both groups, though more prevalent in controls (42.1% in bladder cancer group vs. 56.9% in the control). Other notable species in the bladder cancer group included *Staphylococcus saprophyticus* (11.5%), *Veillonella dispar* (11.4%), *Eggerthella lenta* (11.1%), and *Streptococcus agalactiae* (5.7%). In the control group, in addition to *L. iners*, *Morganella morganii* (9.3%), *Veillonella dispar* (7%), *Bifidobacterium longum* (6.9%), and *Acinetobacter guillouiae* (2.8%) were prominent.

Differential Abundance (LEfSe): LEfSe (linear discriminant analysis effect size) analysis (LDA threshold value > 2, *p* < 0.05) was used to determine significant bacterial compositions between the groups. The LEfSe analysis results of bladder cancer and healthy controls are shown in Figure 1.

**Figure 4:**
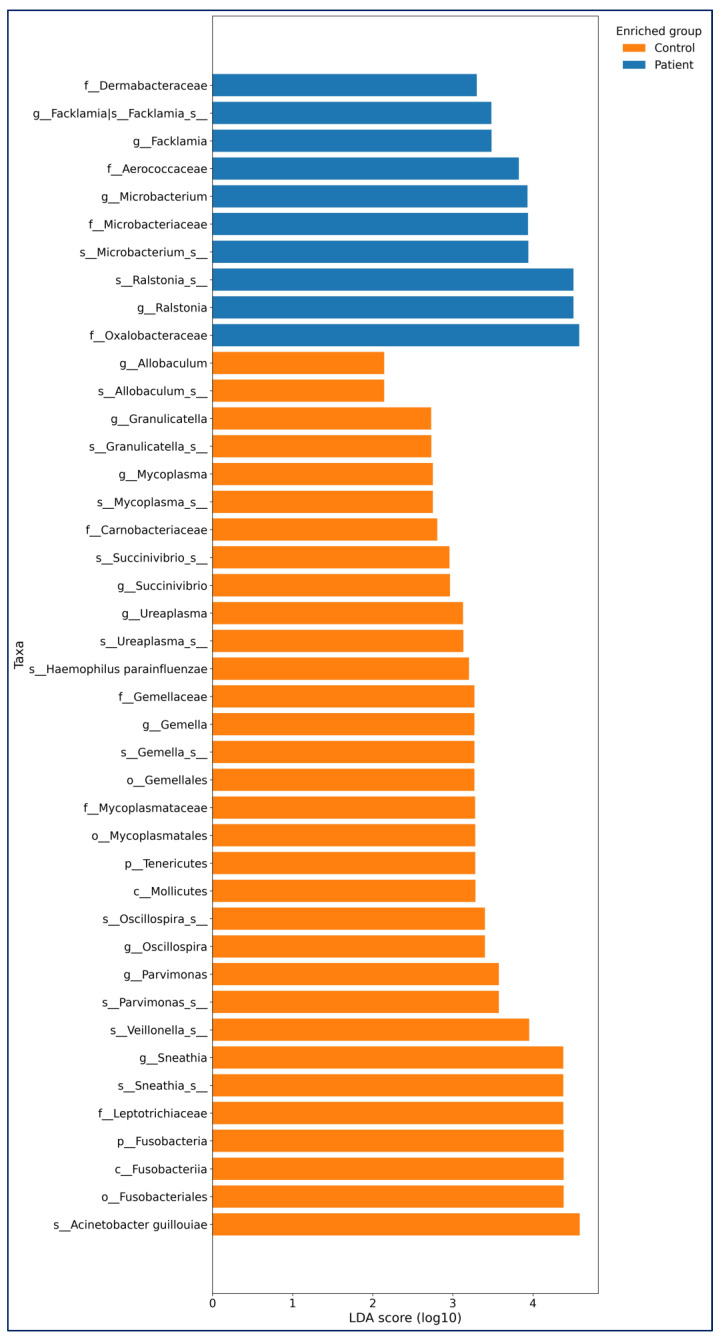
LEfSe (Linear Discriminant Analysis Effect Size) bar plot of differentially abundant urinary microbiota taxa between patients with bladder cancer and healthy controls. Taxa enriched in the Control group are displayed with orange bars and taxa enriched in the Patient group with blue bars. Within each group, taxa are ordered from highest to lowest LDA score (log 10). Taxonomic prefixes: p—phylum, c—class, o—order, f—family, g—genus, s—species.

In the Bladder Cancer group, *Ralstonia* (*p* = 0.036), *Microbacterium* (*p* = 0.046), *and Facklamia* (*p* = 0.017) were more abundant at the genus and species levels. In the control group, genera such as *Sneathia* (*p* = 0.001), *Parvimonas* (*p* < 0.0001), *Oscillospira* (*p* = 0.044), *Gemella* (*p* < 0.001), *Ureaplasma* (*p* < 0.016), *Succinivibrio* (*p* = 0.020), *Mycoplasma* (*p* = 0.004), *Granulicatella* (*p* = 0.023), and *Allobaculum* (*p* = 0.015) were more abundant (Figure 4).

### 3.2. MIBC vs. NMIBC Comparison

**Figure 5:**
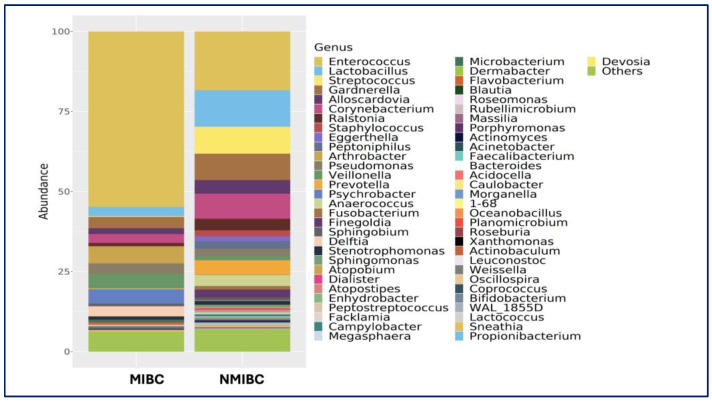
Genus-level relative abundance profiles of urinary microbiota in patients with muscle-invasive bladder cancer (MIBC) and non-muscle-invasive bladder cancer (NMIBC). Stacked bar plots display the proportional distribution of bacterial genera within each group. Only genera with detectable abundance are shown; all remaining low-abundance genera are grouped under “Others”.

No significant differences were observed in alpha or beta diversity between MIBC and NMIBC groups (*p* > 0.05). However, compositional differences were noted (Figure 2). In, MIBC Samples were dominated by *Enterococcus* (58.0%), followed by *Arthrobacter*, *Psychrobacter*, and *Veillonella*. At the species level, *Veillonella dispar* (70.9%) was highly abundant, followed by *Lactobacillus helveticus* (11.3%), *Lactobacillus iners* (6.0%) (Figure 5).

Among NMIBC urinary samples are more taxonomically diverse, *Enterococcus* (26.9%) was the most frequently detected, followed by *Lactobacillus* (12.0%), *Gardnerella* (10.0%), *Streptococcus* (9.4%), with notable abundance of *Lactobacillus iners* (46.7%), *Staphylococcus saprophyticus* (12.9%), and *Eggerthella lenta*, (12.4%), and *Streptococcus agalactiae* (6.3%).

**Figure 6:**
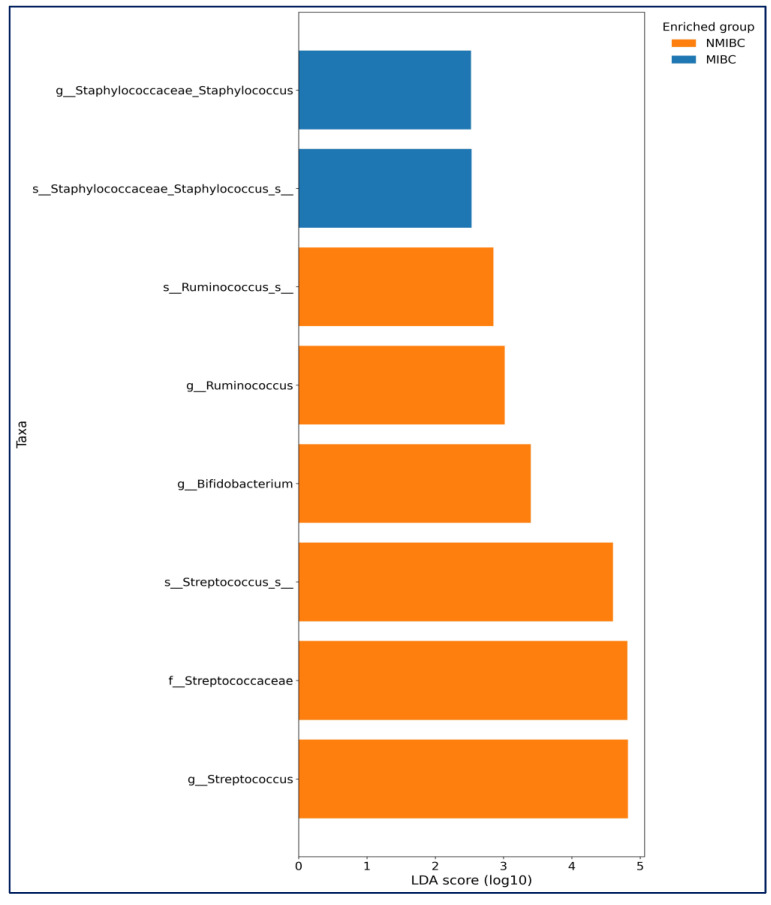
LEfSe analysis identifying bacterial taxa that were significantly different in abundance between muscle-invasive bladder cancer (MIBC) and non-muscle-invasive bladder cancer (NMIBC). Only taxa with an LDA effect size > 2.0 and FDR-corrected *p* < 0.05 are shown. Horizontal bars represent log10-transformed LDA scores, indicated by vertical dotted reference lines. Colors correspond to the enriched groups: NMIBC (orange) and MIBC (blue). Taxonomic prefixes: c—class, o—order, f—family, g—genus, s—species.

LEfSe analysis further showed in MIBC group, Staphylococcus (genus) and *Staphylococcus_s* (species) (*p* = 0.039 for both) were more abundant. In the NMIBC group, *Streptococcus*, *Bifidobacterium*, and *Ruminococcus* (*p* = 0.018, *p* = 0.041 and *p* = 0.043 consecutively) were more abundant (Figure 6).

## 4. Discussion

The alteration of the microbiota has been reported to possibly cause various cancers [15]. Emerging evidence suggests that urinary microbiota may play a role in the development and progression of bladder cancer [11,12,13,14]. Our study investigated the urinary microbial composition in newly diagnosed patients with treatment-naïve bladder cancer compared to healthy controls, with additional subgroup analysis for MIBC vs. NMIBC. While alpha and beta diversity did not differ significantly between groups, distinct taxonomic shifts were observed.

Several earlier studies have reported varying results regarding urinary microbiome diversity in bladder cancer [14]. While some report increased diversity in patients with bladder cancer [16,17,18], especially in NMIBC cases; others, such as Chipollini et al. [19]., observed decreased diversity. In our cohort, we found no significant differences in alpha or beta diversity between patients and controls—a result consistent with Bučević Popović et al. [20] who reported similar findings using midstream urine. For instance, research utilizing 2bRAD-M sequencing identified 527 species, noting a significant reduction in microbial diversity in MIBC tissues compared to NMIBC tissues [21].

At the phylum level, we found that *Firmicutes* was predominant in both groups (63.3% in bladder cancer vs. 46.3% in controls), consistent with Mansour et al. [22] and Bučević Popović et al. [20], who also identified *Firmicutes* dominance in both urine and tissue samples. In contrast, Proteobacteria was higher in controls (39.0%) than in patients with cancer (16.0%). This diverges from studies by Liu et al. [23], and Wu et al. [17], which found Proteobacteria enrichment in tumor tissue. These inconsistencies may reflect geographical, dietary, and sampling differences, underscoring the need for methodological standardization in urinary microbiome research.

At the genus level, our findings reinforce previous suggestions of a potential role for *Enterococcus* in bladder cancer. We observed a higher prevalence of Enterococcus in the cancer group (35.0% vs. 19.8% in controls), and especially in MIBC cases (58.0%). This agrees with findings by Parra-Grande et al. [24], who noted an enrichment of Enterococcus in low-grade tumor mucosa. Given its ability to generate extracellular superoxide and induce DNA damage *Enterococcus* may contribute to carcinogenesis via chronic inflammation and oxidative stress [23,24], In our study, in the bladder cancer group, *Ralstonia*, were enriched at the genus and species levels. Enrichment of *Ralstonia* species (e.g., *R. pickettii*, *R. mannitolilytica*) have been previously described, especially in MIBC cases [25].

In contrast, the *Lactobacillus* genus—known for its protective immunomodulatory and anti-inflammatory properties—was lower in patients with bladder cancer (9.5%) compared to controls (19.8%), particularly in MIBC cases. The dominance of *Lactobacillus* may be protective against the development of bladder cancer as well as against invasive disease. Similar reductions in *Lactobacillus* have been linked to dysbiosis in other mucosal cancers, such as cervical and colorectal cancer [20,26].

We also observed notable findings for *Veillonella dispar*, which was highly enriched in patients with MIBC (70.9%) compared to NMIBC (3.9%). This dramatic increase is consistent with Oresta et al. [26] who associated Veillonella with high-grade bladder tumors. The anaerobic and pro-inflammatory nature of this taxon may reflect a microenvironment conducive to tumor progression. The relative dominance of *Veillonella dispar* in the presence of high-grade bladder cancer suggests that it could be used to predict the course of the disease.

The detection of *Facklamia* exclusively in patients with bladder cancer—and not in controls—further supports its possible role as a novel biomarker. While limited evidence exists, Bučević Popović et al. [20] similarly identified *Facklamia* as enriched in patients with NMIBC. Given its pathogenic potential and absence from the control group’s microbiota, its presence in bladder cancer samples warrants further investigation.

This study has some limitations, including the modest sample size, particularly within the MIBC subgroup, and the absence of functional or metagenomic profiling to explore mechanistic pathways. Because this study was exploratory in nature, no formal power calculation was performed; the sample size was based on feasibility and intended to generate preliminary microbiota profiles for future hypothesis-driven research. Because smoking was significantly more common among patients with bladder cancer and given its known impact on both bladder carcinogenesis and urinary microbiota composition, smoking may represent an important confounder; this limitation has been acknowledged and should be addressed in future studies with adjusted or stratified analyses. Use of midstream urine may introduce urethral microbiota contamination, although standard cleaning procedures were applied. Our use of midstream urine samples aligns with most prior microbiome studies [20,22,26], as this method is practical and reflects bladder-associated microbiota despite the potential for contamination. While catheterized or tissue-derived samples may offer more specificity [23], midstream sampling remains a widely accepted and validated approach, particularly in clinical settings. We did not analyze urine–tissue microbiome correlation, which could clarify site-specific microbial influences. However, inclusion of patients with treatment-naïve bladder cancer minimized confounding from therapeutic interventions (e.g., antibiotics, BCG). We used strict inclusion and exclusion criteria, controlling for age, BMI, recent antibiotic/probiotic use, and immune status. MIBC vs. NMIBC subgroup analysis provided insights into microbial profiles associated with tumor invasiveness.

## 5. Conclusions

While distinct microbial signatures were observed, these findings should be interpreted as exploratory rather than diagnostic, and no biomarker performance characteristics can be inferred from the current dataset. Urinary microbiota alterations may relate to biological processes involved in tumor progression, whereas potential effects on treatment response—such as to intravesical immunotherapy—represent a separate conceptual pathway. 

This study contributes to a growing body of evidence suggesting that urinary microbiota composition differs between patients with bladder cancer and healthy individuals. Notably, higher abundance of *Enterococcus* and *Veillonella dispar*, and reduced *Lactobacillus*, may reflect a shift toward a pro-inflammatory, altered-microbiota environment in bladder cancer, especially in muscle-invasive cases. The exclusive detection of *Facklamia* in patients may also point to its potential use as a non-invasive biomarker. While our findings support the role of urinary dysbiosis in bladder carcinogenesis, larger, prospective, and functionally integrated studies are needed. Longitudinal analyses could reveal whether microbial changes precede tumor development or result from cancer-related alterations. Investigating the microbiota’s role in intravesical therapy response and recurrence may also open new avenues for precision medicine in uro-oncology.

## Figures and Tables

**Table 1:** Baseline demographic and clinical characteristics of the study population.

	Bladder Cancer (n = 30)	Control (n = 20)	*p*-Value
Age (years)	69.4 ± 9.8	67.6 ± 9.3	ns
Gender (Female/Male)	4/26	3/17	ns
Body Mass Index (kg/m^2^)	24.9 ± 3.7	23.8 ± 3.5	ns
Active smoking, n (%)	24 (80%)	9 (45%)	*p* = 0.013
Tumor stage, n (%)			
• pTa	15 (50%)	—	—
• pT1	9 (30%)	—	—
• pT2	6 (20%)	—	—
Tumor grade, n (%)			
• Low grade	12 (40%)	—	—
• High grade	18 (60%)	—	—
Carcinoma in situ present, n (%)	12 (40%)	—	—
Presence of variant histology, n (%)	6 (20%)	—	—

ns: not significant.

## Data Availability

Data available on request from the authors.
